# Query Lifting: Language-integrated query for heterogeneous nested collections

**DOI:** 10.1007/978-3-030-72019-3_21

**Published:** 2021-03-23

**Authors:** Wilmer Ricciotti, James Cheney

**Affiliations:** Imperial College, London, UK; 9 Laboratory for Foundations of Computer Science, University of Edinburgh, Edinburgh, United Kingdom; 10 The Alan Turing Institute, London, United Kingdom

**Keywords:** language-integrated query, nested relations, multisets

## Abstract

Language-integrated query based on comprehension syntax is a powerful technique for safe database programming, and provides a basis for advanced techniques such as *query shredding* or *query flattening* that allow efficient programming with complex nested collections. However, the foundations of these techniques are lacking: although SQL, the most widely-used database query language, supports *heterogeneous* queries that mix set and multiset semantics, these important capabilities are not supported by known correctness results or implementations that assume *homogeneous* collections. In this paper we study language-integrated query for a heterogeneous query language $\mathcal {NRC}_{\lambda }( Set,Bag )$ that combines set and multiset constructs. We show how to normalize and translate queries to SQL, and develop a novel approach to querying heterogeneous nested collections, based on the insight that “local” query subexpressions that calculate nested subcollections can be “lifted” to the top level analogously to lambda-lifting for local function definitions.

## 1 Introduction

Since the rise of relational databases as important software components in the 1980s, it has been widely appreciated that database programming is hard [13]. Databases offer efficient access to flat tabular data using declarative SQL queries, a computational model very different from that of most general-purpose languages. To get the best performance from the database, programmers typically need to formulate important parts of their program's logic as queries, thus effectively programming in two languages: their usual general-purpose language (e.g. Java, Python, Scala) and SQL, with the latter query code typically constructed as unchecked, dynamic strings. Programming in two languages is more than twice as difficult as programming in one language [35]. The result is a hybrid programming model where important parts of the program's functionality are not statically checked and may lead to run-time failures, or worse, vulnerabilities such as SQL injection attacks. This undesirable state of affairs was recognized by Copeland and Maier [13] who coined the term *impedance mismatch* for it.

Though higher-level wrapper libraries and tools such as *object-relational mappings* (ORM) can help ameliorate the impedance mismatch, they often come at a price of performance and lack of transparency, as high-level operations on in-memory objects representing database data are not always mapped efficiently to queries [45]. An alternative approach, which has almost as long a history as the impedance mismatch problem itself, is to elevate queries in the host language from unchecked strings to a typed, domain-specific sublanguage, whose interactions with the rest of the program can be checked and which can be mapped to database queries safely while providing strong guarantees. This approach is nowadays typically called *language-integrated query* following Microsoft's successful LINQ extensions to .NET languages such as C# and F# [36,49]. It is ultimately based on Trinder and Wadler's insight that database queries can be modeled by a form of monadic comprehension syntax [50].

Comprehension-based query languages were placed on strong foundations in the database community in the 1990s [3,4,40,55,33]. A key insight due to Paredaens and van Gucht [40] is that although comprehension-based queries can manipulate nested collections, any expression whose input and output are *flat* collections (i.e. tables of records without other collections nested inside field values) can always be translated to an equivalent query only using flat relations (i.e. can be expressed in an SQL-like language). Wong [55] subsequently generalized this result and gave a constructive proof, in which the translation from nested to flat queries is accomplished through a strongly normalizing rewriting system.

Wong's work has informed a number of successful implementations, such as the influential Kleisli system [56] for biomedical data integration, and the Links programming language [12]. Although the implementation of LINQ in C# and F# was not directly based on normalization, Cheney et al. [7] showed that normalization can be performed as a pre-processing step to improve both reliability and performance of queries, and guarantee that a well-formed query expression evaluates to (at most) one equivalent SQL expression at run time.

Comprehension-based language-integrated query also forms the basis for libraries such as Quill for Scala [41] and Database-Supported Haskell [21]. Most recently, language-integrated query has been extended further to support efficient execution of queries that construct *nested* results [25,8,21,53], by translating such queries to a bounded number of flat queries. This technique, currently implemented in Links and DSH, has several benefits: for example to implement *provenance-tracking* efficiently in queries [17,47]. Fowler et al. [19] showed that in some cases, Links's support for nested query results decreased both the number of queries issued and the total query evaluation time by an order of magnitude or more compared to a Java database application. Unfortunately, there is still a gap between the theory and practice of language-integrated query. Widely-used and practically important SQL features that mix set and multiset collections, such as duplicate elimination, are supported by some implementations, but without guarantees regarding correctness or reliability. So far, such results have only been proved for special cases [7,8], typically for *homogeneous* queries operating on one uniform collection type. For example, in Links, queries have multiset semantics and cannot use duplicate elimination or set-valued operations. To the best of our knowledge the questions of how to correctly translate flat or nested *heterogeneous* queries to SQL are open problems.

In this paper, we solve both open problems. We study a heterogeneous query language $\mathcal{NRC}_\lambda(\mathit{Set}, \mathit{Bag})$, which was introduced and studied in our recent work [42]. We have previously extended the key results on query normalization to $\mathcal{NRC}_\lambda(\mathit{Set}, \mathit{Bag})$ [43], but unlike the homogeneous case, the resulting normal forms do not directly correspond to SQL. In this paper, we first show how flat $\mathcal{NRC}_\lambda(\mathit{Set}, \mathit{Bag})$ queries can be translated to SQL, and we then develop a new approach for evaluating queries over nested heterogeneous collections. The key (and, to us at least, surprising) insight is to recognize that these two subproblems are really just different facets of one problem. That is, when translating flat $\mathcal{NRC}_\lambda(\mathit{Set}, \mathit{Bag})$ queries to SQL, the main obstacle is how to deal with query expressions that depend on local variables; when translating nested $\mathcal{NRC}_\lambda(\mathit{Set}, \mathit{Bag})$ queries to equivalent flat ones, the main obstacle is also how to deal with query expressions that depend on local variables. We solve this problem by observing that such query subexpressions can be *lifted*, analogously to *lambda-lifting* of local function definitions in functional programming [30], by abstracting over their free variables. Differently to lambda-lifting, however, we lift such expressions by converting them to *tabular functions*, or *graphs*, which can be calculated using database query constructs.

The remainder of this paper presents our contributions as follows:

- In section 2 we review the most relevant prior work and present our approach at a high, and we hope accessible, level.
- In sections 3 and 4 we present the core languages $\mathcal{NRC}_\lambda(\mathit{Set}, \mathit{Bag})$ and $\mathcal{NRC}_\mathcal{G}$ which will be used in the rest of the paper.
- Section 5 presents our results on translation of flat $\mathcal{NRC}_\lambda(\mathit{Set}, \mathit{Bag})$ queries to SQL, via $\mathcal{NRC}_\mathcal{G}$.
- Section 6 presents our results on translation of $\mathcal{NRC}_\lambda(\mathit{Set}, \mathit{Bag})$ queries that construct nested results to a bounded number of flat $\mathcal{NRC}_\mathcal{G}$ queries.
- Sections 7 and 8 discuss related work and conclude.

## 2 Overview

In this section we sketch our approach. We use Links syntax [12], which differs in superficial respects from the core calculus in the rest of the paper but is more readable. We rely without further comment on existing capabilities of language-integrated query in Links, which are described elsewhere [11,34,8]. Suppose, hypothetically, we are interested in certain presidential candidates and prescription drugs they may be taking[3]. In Links, an expression querying a small database of presidential candidates and their drug prescriptions can be written as follows:

[3] For example, to see whether drug interactions might explain erratic behavior such as rage tweeting, creeping authoritarianism, or creepiness more generally.

*Cand*

| name | cid |
|---|---|
| DJT | 45 |
| JRB | 46 |

*Pres*

| cid | did | day |
|---|---|---|
| 45 | 101 | Mon |
| 45 | 223 | Tue |
| 45 | 223 | Thu |
| 46 | 765 | Fri |

*Drug*

| did | drug |
|---|---|
| 101 | hydrochloroquine |
| 223 | adderall |
| 765 | caffeine |

$Q_F$

| in | out |
|---|---|
| (DJT,45) | hydrochloroquine |
| (DJT,45) | adderall |
| (JRB,46) | caffeine |

$Q_1$

| name | drug |
|---|---|
| DJT | hydrochloroquine |
| DJT | adderall |
| JRB | caffeine |

**Fig. 1.** Input tables *Cand*, *Pres*, *Drug*, intermediate result of $Q_F$ and result of $Q_1$.

```
Q0 = for (c <- Cand, p <- Pres, d <- Drug)
     where (c.cid == p.cid && p.did == d.did)
     [(name=c.name,drug=d.drug)]
```

Some (totally fictitious and not legally actionable) example data is shown in Figure 1; note that the prescriptions table *Pres* is a multiset containing duplicate entries. Executing this query in Links results in the following SQL query:

```
SELECT c.name, d.drug
FROM Cand c, Pres p, Drug d
WHERE c.cid = p.cid AND p.did = d.did
```

In Links, query results from the database are mapped back to list values nondeterministically, and the result of the above query $Q_0$ will be a list containing two copies of the tuple (`DJT`, `adderall`) and one copy of each of the tuples (`DJT`, `hydrochloroquine`) and (`JRB`, `caffeine`). If we are just interested in which candidates take which drugs and not how many times each drug was taken, we want to remove these duplicates. This can be accomplished in a basic SQL query using the `DISTINCT` keyword after `SELECT`. Currently, in Links there is no way to generate queries involving `DISTINCT`, and this duplicate elimination can only be performed in-memory. While this is not hard to do when the duplicate elimination happens at the end of the query, it is not as clear how to handle deduplication operations correctly in arbitrary places inside queries. Furthermore, SQL has several other operations that can have either set or multiset semantics such as `UNION` and `EXCEPT`: how should they be handled?

To study this problem we introduced a core calculus $\mathcal{NRC}_\lambda(Set, Bag)$ [42] (reviewed in the next section) in which there are two collection types, sets and multisets (or *bags*); duplicate elimination maps a multiset to a set with the same elements, and *promotion* maps a set to the least multiset with the same elements.

We considered, but were not previously able to solve, two problems in the context of $\mathcal{NRC}_\lambda(Set, Bag)$ which are addressed in this paper. First, the fundamental results regarding normalization and translation to SQL have been studied only for *homogeneous* query languages with collections consisting of either sets, bags, or lists. We recently extended the normalization results to $\mathcal{NRC}_\lambda(Set, Bag)$ [43], but the resulting normal forms do not correspond directly to SQL queries if operations such as deduplication, promotion, or bag difference are present. Second, query expressions that construct nested collections cannot be translated directly to SQL and can be very expensive to execute in-memory using nested loops, leading to the $N+1$ *query problem* (or *query avalanche problem* [26]) in which one query is performed for the outer loop and then another $N$ queries are performed, one per iteration of the inner loop. Some techniques have been developed for translating nested queries to a fixed number of flat queries, but to date they either handle only homogeneous set or bag collections [54,8], or lack detailed correctness proofs [26,52].

Regarding the first problem, the closest work in this respect is by Libkin and Wong [33], who studied and related the expressiveness of comprehension-based homogeneous set and bag query languages but did not consider their heterogeneous combination or translation to SQL. The following query illustrates the fundamental obstacle:

```
Q1 = for (c <- Cand)
     for (d <- dedup(for (p <- Pres, d <- Drug)
                       where (c.cid == p.cid && p.did == d.did)
                       [d.drug]))
     [(name=c.name, drug=d)]
```

This query is similar to $Q_0$, but eliminates duplicates among the drugs for each candidate. The query contains a duplicate elimination operation (`dedup`) applied to another query subexpression that refers to $c$, which is introduced in an earlier generator. This is not directly supported in classic SQL: by default the subqueries in `FROM` clauses cannot refer to tuple variables introduced by earlier parts of the `FROM` clause. In fact, this query is expressible in SQL:1999 using the `LATERAL` keyword, which does allow such sideways information-passing:

```
SELECT c.name,d.drug
FROM Cand c, LATERAL (SELECT DISTINCT d.drug
                       FROM Pres p, Drug d
                       WHERE p.cid = c.cid AND p.did = d.did) d
```

(Without the `LATERAL` keyword, this query is not well-formed SQL.) However, such queries have only recently become widely supported, so are not available on legacy databases, and even when supported, are not typically optimized effectively; for example PostgreSQL will evaluate it as a nested loop, with quadratic complexity or worse.

Regarding the second problem, Van den Bussche [54] showed that any query returning nested set collections can be simulated by $n$ flat queries, where $n$ is the number of occurrences of the set collection type in the result. However, this translation has not been used as the basis for a practical system to our knowledge, and does not respect multiset semantics. Cheney et al. [8] provided an analogous *shredding* translation for nested multiset queries, but translated to a richer target language (including SQL:1999 features such as `ROW_NUMBER`) and did not handle operations such as multiset difference or duplicate elimination. Thus, neither approach handles the full expressiveness of a heterogeneous query language over bags and sets. The following query illustrates the fundamental obstacle:

```
Q2 = for (x <- Cand)
     [(name=x.name, drugs=dedup(for (p <- Pres, d <- Drug)
                             where (x.cid == p.cid and p.did == d.did)
                             [d.drug]))]
```

Much like $Q_1$, $Q_2$ builds a multiset of pairs $(name, drugs)$ but here *drugs* is a *set* of all of the drugs taken by candidate *name*. Such a query is, of course, not even syntactically expressible in SQL because it returns a nested collection; it is not expressible in previous work on nested query evaluation either, because the result is a multiset of records, one component of which is a set.

We will now illustrate how to translate $Q_1$ to a plain SQL query (not using LATERAL), and how to translate $Q_2$ to two flat queries such that the nested result can be constructed easily from their flat results. First, note that we can rewrite both queries as follows, introducing an abbreviation $F(x)$ for a query subexpression parameterized by $x$:

```
F(x) = for (p <- Pres, d <- Drug)
       where (x.cid == p.cid and p.did == d.did)
       [d.drug]
Q1   = for (c <- Cand) for (d <- dedup(F(c))) [(name=c.name, drug=d)]
Q2   = for (c <- Cand) [(name=c.name, drugs=dedup(F(c)))]
```

Next, observe that the set of all possible values for $x$ appearing in some call to $F(x)$ is finite, and can even be computed by a query. Therefore, we can write a *closed* query $Q_F$ that builds a lookup table that calculates the *graph* of $F$ (or at least, as much of it as is needed to evaluate the queries) as follows:

```
Q_F = dedup(for (x <- Cand, y <- F(x)) [(in=x,out=y))]
```

Notice that the use of deduplication here is really essential to define $Q_F$ correctly: if we did not deduplicate then there would be repeated tuples in $Q_F$, leading to incorrect results later. If we inline and simplify $F(x)$ in the above query, we get the following:

```
Q_F' = dedup(for (x <- Cand, y <- Pres, z <- Drug)
             where (x.cid == y.cid && y.did = z.did)
             [(in=x,out=z.drug)])
```

Finally we may replace the call to $F(x)$ in $Q_1$ with a lookup to $Q'_F$, as follows:

```
Q1' = for (c <- Cand, f <- Q_F') where (c == f.in)
      [(name=c.name, drug=f.out)]
```

This expression may now be translated directly to SQL, because the argument to `dedup` is now closed:

```
SELECT c.name,f.drug
FROM Cand c, (SELECT DISTINCT x.name,x.cid,z.drug
              FROM Cand x, Pres y, Drug z
              WHERE x.cid = y.cid AND y.did = z.did) f
WHERE c.cid = f.cid AND c.name = f.name
```

$Q_{21}$

| name | drugs |
|---|---|
| DJT | (DJT,45) |
| JRB | (JRB,46) |

$Q_{22}$

| in | out |
|---|---|
| (DJT,45) | hydrochloroquine |
| (DJT,45) | adderall |
| (JRB,46) | caffeine |

$Q_2$

| name | drugs |
|---|---|
| DJT | {hydrochloroquine, adderall} |
| JRB | {caffeine} |

**Fig. 2.** Intermediate results of $Q_{21}, Q_{22}$ and result of $Q_2$.

Although this query looks a bit more complex than the one given earlier using `LATERAL`, it can be optimized more effectively, for example PostgreSQL generates a query plan that uses a hash join, giving quasi-linear complexity.

On the other hand, to deal with $Q_2$, we refactor it into two closed, flat queries $Q_{21}, Q_{22}$ and an expression $Q_2'$ that builds the nested result from their flat results (illustrated in Figure 2):

```
Q_21 = for (x <- Cand) [(name=x.name, drugs=x)]
Q_22 = Q_F
Q2'  = for (x <- Q21)
        [(name=x.name,
          drugs=for (y <- Q_22) where(x.drugs == y.in) [y.out])]
```

Notice that in $Q_{21}$ we replaced the call to $F$ with the argument $x$, while $Q_{22}$ is just $Q_F$ again. The final expression $Q_2'$ builds the nested result (in the host language's memory) by traversing $Q_{21}$ and computing the set value of each *cs* field by looking up the appropriate values from $Q_{22}$. Thus, the original query result can be computed by first evaluating $Q_{21}$ and $Q_{22}$ on the database, and then evaluating the final *stitching* query expression in-memory. (In practice, as discussed in Cheney et al. [8], it is important for performance to use a more sophisticated stitching algorithm than the above naive nested loop, but in this paper we are primarily concerned with the correctness of the transformation.)

The above examples are a bit simplistic, but illustrate the key idea of *query lifting*. In the rest of this paper we place this approach on a solid foundation, and (partially inspired by Gibbons et al. [20]), to help clarify the reasoning we extend the calculus with a type of *tabulated functions* or *graphs* $\overrightarrow{\sigma} \dashrightarrow \{\tau\}$, with *graph abstraction* introduction form $\mathcal{G}(-;-)$ and *graph application* $M \circledast \langle\overrightarrow{x}\rangle$. In our running example we could define $Q_F = \mathcal{G}(x \leftarrow R; F(x))$, and we would use the application operation $M \circledast \langle\overrightarrow{x}\rangle$ to extract the set of elements corresponding to $x$ in $Q_F$. We will also consider tabular functions that return multisets rather than sets, in order to deal with queries that return nested multisets.

## 3 Background

We recap the main points from [42], which introduced a calculus $\mathcal{NRC}_\lambda(\mathit{Set}, \mathit{Bag})$ with the following syntax:

$$
\begin{array}{lrl}
\textbf{Types} & \sigma,\tau ::= & b \mid \langle\overrightarrow{\ell:\sigma}\rangle \mid \{\sigma\} \mid \{\!|\sigma|\!\} \mid \sigma\to\tau \\
\textbf{Terms} & M,N ::= & x \mid t \mid c(\overrightarrow{M}) \mid \langle\overrightarrow{\ell=M}\rangle \mid M.\ell \mid \lambda x.M \mid M\;N \\
& \mid & \emptyset \mid \{M\} \mid M\cup N \mid \bigcup\{M|\Theta\} \\
& \mid & \mho \mid \{\!|M|\!\} \mid M\uplus N \mid M-N \mid \biguplus\{\!|M|\Theta|\!\} \\
& \mid & \delta M \mid \iota M \mid M\ \textbf{where}_{\mathsf{set}}\ N \mid M\ \textbf{where}_{\mathsf{bag}}\ N \\
& \mid & \textbf{empty}_{\mathsf{set}}(M) \mid \textbf{empty}_{\mathsf{bag}}(M) \\
\textbf{Generators} & \Theta ::= & \overrightarrow{x\leftarrow M}
\end{array}
$$

We distinguish between (local) variables $x$ and (global) table names $t$, and assume standard primitive types $b$ and primitive operations $c(\overrightarrow{M})$ including respectively Booleans $\mathbf{B}$ and equality at every base type. The syntax for records and record projection $\langle\overrightarrow{\ell=M}\rangle, M.\ell$, and for lambda-abstraction and application $\lambda x.M, M\;N$ is standard; as usual, let-binding is definable. Set operations include empty set $\emptyset$, singleton construction $\{M\}$, union $M\cup N$, one-armed conditional $M$ **where**$_{\mathsf{set}}$ $N$, emptiness test $\textbf{empty}_{\mathsf{set}}(M)$, and comprehension $\bigcup\{M \mid \Theta\}$, where $\Theta$ is a sequence of generators $x\leftarrow M$. Similarly, multiset operations include empty bag $\mho$, singleton $\{\!|M|\!\}$, bag union $M\uplus N$, bag difference $M-N$, conditional $M$ **where**$_{\mathsf{bag}}$ $N$, emptiness test $\textbf{empty}_{\mathsf{bag}}(M)$. The syntax is completed by duplicate elimination $\delta M$ (converting a bag $M$ into a set with the same object type) and promotion $\iota M$ (which produces the bag containing all the elements of the set $M$, with multiplicity 1).

The one-way conditional operations $M$ **where**$_{\mathsf{set}}$ $N$ and $M$ **where**$_{\mathsf{bag}}$ $N$ evaluate Boolean test $N$, and return collection $M$ if $N$ is true, otherwise the empty set/bag; two-way conditionals can supported without problems. Other set operations, such as intersection, membership, subset, and equality are also definable, as are bag operations such as intersection [4,33]. Also, we may define $\textbf{empty}_{\mathsf{bag}}(M)$ as $\textbf{empty}_{\mathsf{set}}(\delta(M))$ and $M$ **where**$_{\mathsf{set}}$ $N$ as $\delta(\iota(M)\ \textbf{where}_{\mathsf{bag}}\ N)$, but we prefer to include these constructs as primitives for symmetry. Generally, we will allow ourselves to write $M$ **where** $N$ and $\textbf{empty}(M)$ without subscripts if the collection kind of these operations is irrelevant or made clear by the context. We freely use syntax for unlabeled tuples $\langle\overrightarrow{M}\rangle, M.i$ and tuple types $\overrightarrow{\sigma}$ and consider them to be syntactic sugar for labeled records.

The typing rules for the calculus are standard and provided in the full version of this paper [44]. For the purposes of this discussion, we will highlight two features of the type system. The first is that the calculus used here differs from our previous work by using constants and table names, whose types are described by a fixed signature $\Sigma$:

$$
\frac{\Sigma(c) = \overrightarrow{b}\to b \qquad (\Gamma\vdash M_i:\sigma_i)_{i=1,\dots,n}}{\Gamma\vdash c(\overrightarrow{M}):\tau}
\qquad\qquad
\frac{\Sigma(t)=\overrightarrow{\ell:b}}{\Gamma\vdash t:\{\!|\langle\overrightarrow{\ell:b}\rangle|\!\}}
$$

As usual, a typing judgment $\Gamma\vdash M:\sigma$ states that a term $M$ is well-typed of type $\sigma$, assuming that its free variables have the types declared in the typing context $\Gamma = x_1:\sigma_1,\dots,x_k:\sigma_k$. For the two rules above, note in particular that the primitive functions $c$ can only take inputs of base type and produce results at base type, and table constants $t$ are always multisets of records where the fields are of base type. We refer to a type of the form $\langle\overrightarrow{\ell : b}\rangle$ as *flat*; if $\sigma$ is flat, we refer to $\{\sigma\}$ and $\lbag\sigma\rbag$ as *flat collection types*.

The second is that our type system uses an approach à la Church, meaning that variable abstractions (in lambdas/comprehensions), empty sets and empty bags are annotated with their type in order to ensure the uniqueness of typing.

**Lemma 1.** *In $\mathcal{NRC}_\lambda(Set, Bag)$, if $\Gamma \vdash M : \sigma$ and $\Gamma \vdash M : \tau$, then $\sigma = \tau$.*

In the context of a larger language implementation, most of these type annotations can be elided and inferred by type inference. We have chosen to dispense with these details in the main body of this paper to avoid unnecessary syntactic cluttering.

We will use a largely standard denotational semantics for $\mathcal{NRC}_\lambda(Set, Bag)$, in which sets and multisets are modeled as finitely-supported functions from their element types to Boolean values $\{0, 1\}$ or natural numbers respectively. This approach follows the so-called $K$-relation semantics for queries [23,18] as used for example in the HoTTSQL formalization [10]. The full typing rules and semantics are included in the full version of this paper [44].

$\mathcal{NRC}_\lambda(Set, Bag)$ subsumes previous systems including $\mathcal{NRC}$ [4,55], $\mathcal{BQL}$ [33] and $\mathcal{NRC}_\lambda$ [11,8]. In this paper, we restrict our attention to queries in which collection types taking part in $\delta$, $\iota$ or bag difference contain only flat records. There are various reasons for excluding function types from these operators: for starters, any concrete implementation that used function types in these positions would need to decide the equality of functions; secondly, our rewrite system can ensure that a term whose type does not contain function types has a normal form without lambda abstractions and applications only if any $\delta$, $\iota$, or bag difference used in that term are applied to first-order collections. We thus want to exclude terms such as:

$$\biguplus \lbag x\ \lbag 1 \rbag\ \lbag 2 \rbag | x \leftarrow \iota(\{\lambda yz.y\} \cup \{\lambda yz.z\}) \rbag$$

which do not have an SQL representation despite having a flat collection type.

In order to obtain simpler normal forms, in which comprehensions only reference generators with a flat collection type, we also disallow nested collections within $\delta$, $\iota$, and bag difference. We believe this is without loss of generality because of Libkin and Wong's results showing that allowing such operations at nested types does not add expressiveness to $\mathcal{BQL}$.

We have extended Wong's normalizing rewrite rule system, so as to simplify queries to a form that is close to SQL, with no intermediate nested collections. Since our calculus is more liberal than Wong's, allowing queries to be defined by mixing sets and bags and also using bag difference, we have added non-standard rules to take care of unwanted situations. In particular, we use the following constrained eta-expansions for comprehensions:

$$\bigcup\{\delta(M - N) | \Theta\} \leadsto \bigcup\{\{z\} | \Theta, z \leftarrow \delta(M - N)\}$$

$$\biguplus \lbag \iota M | \Theta \rbag \leadsto \biguplus \lbag \lbag z \rbag | \Theta, z \leftarrow \iota M \rbag$$

$$\biguplus \lbag M - N | \Theta \rbag \leadsto \biguplus \lbag \lbag z \rbag | \Theta, z \leftarrow M - N \rbag$$

| | |
|---|---|
| **General normal forms** | $M ::= X \mid \langle\overrightarrow{\ell = M}\rangle \mid Q \mid R$ |
| **Base type terms** | $X ::= x.\ell \mid c(\overrightarrow{X}) \mid \mathbf{empty}_{\mathsf{set}}(Q^*) \mid \mathbf{empty}_{\mathsf{bag}}(R^*)$ |
| **Set normal forms** | $Q ::= \bigcup \overrightarrow{C}$ |
| | $C ::= \bigcup\{\{M\}\ \mathbf{where}_{\mathsf{set}}\ X \vert \overrightarrow{x \leftarrow F}\}$ |
| | $F ::= \delta t \mid \delta(R_1^* - R_2^*)$ |
| **Bag normal forms** | $R ::= \biguplus \overrightarrow{D}$ |
| | $D ::= \biguplus\wr\wr M \wr\ \mathbf{where}_{\mathsf{bag}}\ X \vert \overrightarrow{x \leftarrow G}\wr$ |
| | $G ::= t \mid \iota Q^* \mid R_1^* - R_2^*$ |

**Fig. 3.** Nested relational normal forms.

The rationale of these rules is that in order to achieve, for comprehensions, a form that can be easily translated to an SQL select query, we need to move all the syntactic forms that are blocking to most normalization rules (i.e. promotion and bag difference) from the head of the comprehension to a generator. In order for this strategy to work out, we also need to know that the type of these subexpressions is flat, as we previously mentioned.

In Figure 3 we show the grammar for the normal forms for terms of *nested relational types*, i.e. types of the following form:

$$\sigma ::= b \mid \langle\overrightarrow{\ell : \sigma}\rangle \mid \{\sigma\} \mid \wr\sigma\wr$$

For ease of presentation, the grammar actually describes a "standardized" version of the normal forms in which:

- $\emptyset$ is represented as the trivial union $\bigcup \overrightarrow{C}$ where $\overrightarrow{C}$ is the empty sequence; $\mho$ has a similar representation using a trivial disjoint union;
- comprehensions without a guard are considered to be the same as those with a trivial $\mathsf{true}$ guard:

$$\bigcup\{\{M\} \vert \Theta\} = \bigcup\{\{M\}\ \mathbf{where}\ \mathsf{true} \mid \Theta\}$$

- singletons that do not appear as the head of a comprehension are represented as trivial comprehensions:

$$\{M\} = \bigcup\{\{M\} \mid \ \}$$

Each normal form $M$ can be either a term of base type $X$, a tuple $\langle\overrightarrow{\ell = M}\rangle$, a set $Q$, or a bag $R$. The normal forms of sets and bags are rather similar, both being defined as unions of comprehensions with a singleton head. The generators for set comprehensions $F$ include deduplicated tables and deduplicated bag differences; the generators for bag comprehensions $G$ must be either tables, promoted set queries, or bag differences.

The non-terminals used as the arguments of emptiness tests, promotion, and bag difference have been marked with a star to emphasize the fact that they

$$
\begin{array}{rclrcl}
(\emptyset)^{\mathsf{sql}} &=& \texttt{SELECT}\ 42\ \texttt{WHERE}\ 0 = 1 & (\mho)^{\mathsf{sql}} &=& \texttt{SELECT}\ 42\ \texttt{WHERE}\ 0 = 1 \\
(x.\ell)^{\mathsf{sql}} &=& x.\ell & (c(\overrightarrow{X}))^{\mathsf{sql}} &=& (c)^{\mathsf{sql}}(\overrightarrow{(X)^{\mathsf{sql}}}) \\
(\langle\overrightarrow{\ell = X}\rangle)^{\mathsf{sql}} &=& (X_1)^{\mathsf{sql}}\ \texttt{AS}\ \ell_1, \ldots, (X_n)^{\mathsf{sql}}\ \texttt{AS}\ \ell_n && & \\
(\mathbf{empty}_{\mathsf{set}}(Q^*))^{\mathsf{sql}} &=& \texttt{NOT EXISTS}\ (Q^*)^{\mathsf{sql}} & (\mathbf{empty}_{\mathsf{bag}}(R^*))^{\mathsf{sql}} &=& \texttt{NOT EXISTS}\ (R^*)^{\mathsf{sql}} \\
(Q_1^* \cup Q_2^*)^{\mathsf{sql}} &=& (Q_1^*)^{\mathsf{sql}}\ \texttt{UNION}\ (Q_2^*)^{\mathsf{sql}} & (R_1^* \uplus R_2^*)^{\mathsf{sql}} &=& (R_1^*)^{\mathsf{sql}}\ \texttt{UNION ALL}\ (R_2^*)^{\mathsf{sql}} \\
(t)^{\mathsf{sql}} &=& \texttt{SELECT}\ *\ \texttt{FROM}\ t & (R_1^* - R_2^*)^{\mathsf{sql}} &=& (R_1^*)^{\mathsf{sql}}\ \texttt{EXCEPT ALL}\ (R_2^*)^{\mathsf{sql}} \\
(\delta t)^{\mathsf{sql}} &=& \texttt{SELECT DISTINCT}\ *\ \texttt{FROM}\ t & (\iota(Q^*))^{\mathsf{sql}} &=& (Q^*)^{\mathsf{sql}} \\
(\delta(R_1^* - R_2^*))^{\mathsf{sql}} &=& \multicolumn{4}{l}{\texttt{SELECT DISTINCT}\ *\ \texttt{FROM}\ ((R_1^*)^{\mathsf{sql}}\ \texttt{EXCEPT ALL}\ (R_2^*)^{\mathsf{sql}}s)\ r} \\
(x \leftarrow F)^{\mathsf{sql}} &=& \begin{cases} ((F)^{\mathsf{sql}})\ x & (x \text{ closed}) \\ \texttt{LATERAL}\ ((F)^{\mathsf{sql}})\ x & (\text{otherwise}) \end{cases} && & \\
(x \leftarrow G)^{\mathsf{sql}} &=& \begin{cases} ((G)^{\mathsf{sql}})\ x & (x \text{ closed}) \\ \texttt{LATERAL}\ ((G)^{\mathsf{sql}})\ x & (\text{otherwise}) \end{cases} && &
\end{array}
$$

$$(\textstyle\bigcup\{\{M^*\}\ \mathbf{where}_{\mathsf{set}}\ X \mid \overrightarrow{x \leftarrow F}\})^{\mathsf{sql}} = \texttt{SELECT DISTINCT}\ (M^*)^{\mathsf{sql}}\ \texttt{FROM}\ \overrightarrow{(x \leftarrow F)^{\mathsf{sql}}}\ \texttt{WHERE}\ (X)^{\mathsf{sql}}$$

$$(\biguplus\wr\wr M^*\int\ \mathbf{where}_{\mathsf{bag}}\ X \mid \overrightarrow{x \leftarrow G}\int)^{\mathsf{sql}} = \texttt{SELECT}\ (M^*)^{\mathsf{sql}}\ \texttt{FROM}\ \overrightarrow{(x \leftarrow G)^{\mathsf{sql}}}\ \texttt{WHERE}\ (X)^{\mathsf{sql}}$$

**Fig. 4.** Translation to SQL

must have a flat collection type. The corresponding grammar can be obtained from the grammar for nested normal forms by replacing the rule for $M$ with the following:

$$M^* ::= \langle\overrightarrow{\ell = X}\rangle$$

Normalized queries can be translated to SQL as shown in Figure 4 as long as they have a flat collection type. The translation uses `SELECT DISTINCT` and `UNION` where a set semantics is needed, and `SELECT`, `UNION ALL` and `EXCEPT ALL` in the case of bag semantics. Note that promotion expressions $\iota Q^*$ are translated simply by translating $Q^*$, because in SQL there is no type distinction between set and multiset queries: all query results are multisets, and sets are considered to be multisets having no duplicates.

The other main complication in this translation is in handling generators $x \leftarrow F$, $x \leftarrow G$ where $F$ or $G$ may be a non-closed expression $\iota(Q^*)$, $R_1^* - R_2^*$, or $\delta(R_1^* - R_2^*)$ containing references to other locally-bound variables. To deal with the resulting lateral variable references, we add the `LATERAL` keyword to such queries. As explained earlier, the use of `LATERAL` can be problematic and we will return to this issue in Section 5.

*Remark 1 (Record flattening).* The above translations handle queries that take flat tables as input and produce flat results (collections of flat records $\langle\overrightarrow{\ell : b}\rangle$). It is straightforward to support queries that return nested records (i.e. records containing other records, but not collections). For example, a query $M : \wr\langle b_1, \langle b_2, b_3\rangle\rangle\int$ can be handled by defining both directions of the obvious isomorphism $N : \wr\langle b_1, \langle b_2, b_3\rangle\rangle\int \cong \wr\langle b_1, b_2, b_3\rangle\int : N^{-1}$, normalizing the flat query $N \circ M$, evaluating the corresponding SQL, and applying the inverse $N^{-1}$ to the results. Such *record flattening* is described in detail by Cheney et al. [9] and is implemented in Links, so we will use it from now on without further discussion.

$$\frac{(\Gamma, \overrightarrow{x_{i-1} : \sigma_{i-1}} \vdash L_i : \{\sigma_i\})_{i=1,\ldots,n} \qquad \Gamma, \overrightarrow{x : \sigma} \vdash M : \{\tau\}}{\Gamma \vdash \mathcal{G}^{\mathsf{set}}(\overrightarrow{x \leftarrow L}; M) : \vec{\sigma} \rightarrowtail \{\tau\}} \qquad \frac{(\Gamma, \overrightarrow{x_{i-1} : \sigma_{i-1}} \vdash L_i : \{\sigma_i\})_{i=1,\ldots,n} \qquad \Gamma, \overrightarrow{x : \sigma} \vdash M : \Lbag\tau\Rbag}{\Gamma \vdash \mathcal{G}^{\mathsf{bag}}(\overrightarrow{x \leftarrow L}; M) : \vec{\sigma} \rightarrowtail \Lbag\tau\Rbag}$$

$$\frac{\Gamma \vdash M : \vec{\sigma} \rightarrowtail \tau \qquad (\Gamma \vdash N_i : \sigma_i)_i}{\Gamma \vdash M \circledast (\vec{N}) : \tau} \qquad \frac{\Gamma \vdash M : \vec{\sigma} \rightarrowtail \Lbag\tau\Rbag \qquad \Gamma \vdash N : \vec{\sigma} \rightarrowtail \Lbag\tau\Rbag}{\Gamma \vdash M - N : \vec{\sigma} \rightarrowtail \Lbag\tau\Rbag}$$

$$\frac{\Gamma \vdash M : \vec{\sigma} \rightarrowtail \{\tau\} \qquad \Gamma \vdash N : \vec{\sigma} \rightarrowtail \{\tau\}}{\Gamma \vdash M \cup N : \vec{\sigma} \rightarrowtail \{\tau\}} \qquad \frac{\Gamma \vdash M : \vec{\sigma} \rightarrowtail \Lbag\tau\Rbag \qquad \Gamma \vdash N : \vec{\sigma} \rightarrowtail \Lbag\tau\Rbag}{\Gamma \vdash M \uplus N : \vec{\sigma} \rightarrowtail \Lbag\tau\Rbag}$$

$$\frac{\Gamma \vdash M : \vec{\sigma} \rightarrowtail \Lbag\tau\Rbag}{\Gamma \vdash \delta M : \vec{\sigma} \rightarrowtail \{\tau\}} \qquad \frac{\Gamma \vdash M : \vec{\sigma} \rightarrowtail \{\tau\}}{\Gamma \vdash \iota M : \vec{\sigma} \rightarrowtail \Lbag\tau\Rbag}$$

**Fig. 5.** $\mathcal{NRC}_{\mathcal{G}}$ additional typing rules.

## 4 A relational calculus of tabular functions

We now introduce $\mathcal{NRC}_{\mathcal{G}}$, an extension of the calculus $\mathcal{NRC}_{\lambda}(Set, Bag)$ providing a new type of finite tabular function graphs (in the remainder of this paper, also called simply "graphs"; they are similar to the finite maps and tables of Gibbons et al. [20]). The syntax of $\mathcal{NRC}_{\mathcal{G}}$ is defined as follows:

**Types** $\quad \sigma, \tau ::= \cdots \mid \vec{\sigma} \rightarrowtail \tau$

**Terms** $\quad M, N ::= \cdots \mid \mathcal{G}^{\mathsf{set}}(\Theta; N) \mid \mathcal{G}^{\mathsf{bag}}(\Theta; N) \mid M \circledast (\vec{N})$

Semantically, the type of graphs $\vec{\sigma} \rightarrowtail \tau$ will be interpreted as the set of finite functions from sequences of values of type $\vec{\sigma}$ to values in $\tau$: such functions can return non-trivial values only for a finite subset of their input type. In our settings, we will require the output type of graphs to be a collection type (i.e. $\tau$ shall be either $\{\tau'\}$ or $\Lbag\tau'\Rbag$ for some $\tau'$), and we will use $\emptyset$ or $\mho$ as the trivial value. The typing rules involving graphs are shown in Figure 5.

Graphs are created using the *graph abstraction* operations $\mathcal{G}^{\mathsf{set}}(\Theta; N)$ and $\mathcal{G}^{\mathsf{bag}}(\Theta; N)$, where $\Theta$ is a sequence of generators in the form $\overrightarrow{x \leftarrow M}$; the dual operation of *graph application* is denoted by $M \circledast (\vec{N})$. An expression of the form $\mathcal{G}^{\mathsf{set}}(\overrightarrow{x \leftarrow M}; N)$ is used to construct a (finite) tabular function mapping each sequence of values $R_1, \ldots, R_n$ in the sets $M_1, \ldots, M_n$ to the set $N\left[{}^{\vec{R}}/_{\vec{x}}\right]$. If each $M_i$ has type $\{\sigma_i\}$ and $N$ has type $\{\tau\}$, then the graph has type $\vec{\sigma} \rightarrowtail \{\tau\}$. Similarly, if $N$ has type $\Lbag\tau\Rbag$, $\mathcal{G}^{\mathsf{bag}}(\overrightarrow{x \leftarrow M}; N)$ has type $\vec{\sigma} \rightarrowtail \Lbag\tau\Rbag$. The terms $M_1, \ldots, M_n$ constitute the (finite) *domain* of this graph. When the kind of graph application (set-based or bag-based) is clear from the context or unimportant, we will allow ourselves to write $\mathcal{G}(-;-)$ instead of $\mathcal{G}^{\mathsf{set}}(-;-)$ or $\mathcal{G}^{\mathsf{bag}}(-;-)$.

A graph $G$ of type $\overrightarrow{\sigma} \multimap \tau$ can be applied to a sequence of terms $N_1, \ldots, N_n$ of type $\sigma_1, \ldots, \sigma_n$ to obtain a term of type $\tau$. If $G = \mathcal{G}(\overrightarrow{x \leftarrow L}; M)$, then we will want the semantics of $\mathcal{G}(\overrightarrow{x \leftarrow L}; M) \circledast (\overrightarrow{N})$ to be the same as that of $M\left[\overrightarrow{N}/\overrightarrow{x}\right]$, provided that each of the $N_i$ is in the corresponding element of the domain of the graph. The typing rule does *not* enforce this requirement and if any of the $N_i$ is not an element of $L_i$, the graph application will evaluate to an empty set or bag (depending on $\tau$).

Graphs can also be merged by union, using $\cup$ or $\uplus$ depending on their output collection kind. Furthermore, graphs that return bags can be subtracted from one another using bag difference; the deduplication and promotion operations also extend to graphs in the obvious way.

**Lemma 2.** *In $\mathcal{NRC}_{\mathcal{G}}$, $\Gamma \vdash M : \sigma$ and $\Gamma \vdash M : \tau$, then $\sigma = \tau$.*

Whenever $M$ is well typed and its typing environment is made clear by the context, we will allow ourselves to write $ty(M)$ for the type of $M$. Furthermore, given a sequence of generators $\Theta = x_1 \leftarrow L_1, \ldots x_n \leftarrow L_n$, such that for $i = 1, \ldots, n$ we have $x_1 : \sigma_1, \ldots, x_{i-1} : \sigma_{i-1} \vdash L_i : \sigma_i$, we will write $ty(\Theta)$ to denote the associated typing context:

$$ty(\Theta) := x_1 : \sigma_1, \ldots, x_n : \sigma_n$$

## 4.1 Semantics and translation to $\mathcal{NRC}_\lambda(Set, Bag)$

The semantics of $\mathcal{NRC}_\lambda(Set, Bag)$ is extended to $\mathcal{NRC}_{\mathcal{G}}$ as follows:

$$\begin{aligned}
&\left[\!\left[\mathcal{G}^{\mathsf{set}}(\overrightarrow{x \leftarrow L}; M)\right]\!\right] \rho(\overrightarrow{u}, v) \\
&\quad = (\textstyle\bigwedge_i [\![L_i]\!]\, \rho[x_1 \mapsto u_1, \ldots, x_{i-1} \mapsto u_{i-1}]u_i) \wedge [\![M]\!]\, \rho[\overrightarrow{x \mapsto u}]v \\
&\left[\!\left[\mathcal{G}^{\mathsf{bag}}(\overrightarrow{x \leftarrow L}; M)\right]\!\right] \rho(\overrightarrow{u}, v) \\
&\quad = (\textstyle\bigwedge_i [\![L_i]\!]\, \rho[x_1 \mapsto u_1, \ldots, x_{i-1} \mapsto u_{i-1}]u_i) \times [\![M]\!]\, \rho[\overrightarrow{x \mapsto u}]v \\
&\left[\!\left[M \circledast (\overrightarrow{N})\right]\!\right] \rho v = [\![M]\!]\, \rho\ (\overrightarrow{[\![N]\!]\, \rho}, v)
\end{aligned}$$

In this definition, graph abstractions are interpreted as collections of pairs of values $(\overrightarrow{u}, v)$ where the $\overrightarrow{u}$ represent the input and $v$ the corresponding output of the graph; consequently, the semantics of a graph $\mathcal{G}^{\mathsf{set}}(\overrightarrow{x \leftarrow L}; M)$ states that the multiplicity of $(\overrightarrow{u}, v)$ is equal to the multiplicity of $v$ in the semantics of $M$ (where each $x_i$ is mapped to $u_i$) if each $u_i$ is in the semantics of $L_i$, and zero otherwise. The semantics of bag graph abstractions is similar, with $\times$ substituted for $\wedge$ to allow multiplicities greater than one in the graph output.

For graph applications $M \circledast (\overrightarrow{N})$, the multiplicity of $v$ is obtained as the multiplicity of $(\overrightarrow{[\![N]\!]\, \rho}, v)$ in the semantics of $M$. The semantics of set and bag union, bag difference, bag deduplication, and set promotion, as defined in $\mathcal{NRC}_\lambda(Set, Bag)$, are extended to graphs and remain otherwise unchanged in $\mathcal{NRC}_{\mathcal{G}}$.

In fact (as noted for example by Gibbons et al. [20]), the graph constructs of $\mathcal{NRC}_{\mathcal{G}}$ are just a notational convenience: we can translate $\mathcal{NRC}_{\mathcal{G}}$ back to $\mathcal{NRC}_{\lambda}(Set, Bag)$ by translating types $\overrightarrow{\sigma} \multimap \{\tau\}$ and $\overrightarrow{\sigma} \multimap \{\!|\tau|\!\}$ to $\{\langle\overrightarrow{\sigma},\tau\rangle\}$ and $\{\!|\langle\overrightarrow{\sigma},\tau\rangle|\!\}$ respectively, and the term constructs are rewritten as follows:

$$\begin{aligned}
\mathcal{G}^{\mathsf{set}}(\overrightarrow{x \leftarrow L}; M) &\rightsquigarrow \bigcup\{\{\langle\overrightarrow{x}, y\rangle\} \mid \overrightarrow{x \leftarrow L}, y \leftarrow M\} \\
\mathcal{G}^{\mathsf{bag}}(\overrightarrow{x \leftarrow L}; M) &\rightsquigarrow \biguplus\{\!|\{\!|\langle\overrightarrow{x}, y\rangle|\!\} \mid \overrightarrow{x \leftarrow \iota(L)}, y \leftarrow M|\!\} \\
M \circledast \langle\overrightarrow{N}\rangle &\rightsquigarrow \bigcup\{\{y\}\ \mathbf{where}_{\mathsf{set}}\ \overrightarrow{x} = \overrightarrow{N} \mid \langle\overrightarrow{x}, y\rangle \leftarrow M\} \quad (M : \overrightarrow{\sigma} \multimap \{\tau\}) \\
M \circledast \langle\overrightarrow{N}\rangle &\rightsquigarrow \biguplus\{\!|\{\!|y|\!\}\ \mathbf{where}_{\mathsf{bag}}\ \overrightarrow{x} = \overrightarrow{N} \mid \langle\overrightarrow{x}, y\rangle \leftarrow M|\!\} \quad (M : \overrightarrow{\sigma} \multimap \{\!|\tau|\!\})
\end{aligned}$$

## 5 Delateralization

As explained at the end of section 3, if a subexpression of the form $\iota(N)$ or $N_1 - N_2$ contains free variables introduced by other generators in the query (i.e. not globally-scoped table variables), such queries cannot be translated directly to SQL, unless the SQL:1999 `LATERAL` keyword is used.

More precisely, we can give the following definition of lateral variable occurrence.

**Definition 1.** *Given a query containing a comprehension* $\bigcup\{M \mid \Theta, x \leftarrow N, \Theta'\}$ *or* $\biguplus\{\!|M \mid \Theta, x \leftarrow N, \Theta'|\!\}$ *as a subterm, we say that* $x$ occurs laterally *in* $\Theta'$ *if, and only if, there is a binding* $y \leftarrow N'$ *in* $\Theta'$ *such that* $x \in \mathrm{FV}(N')$.

Since `LATERAL` is not implemented on all databases, and is sometimes implemented inefficiently, we would still like to avoid it. In this section we show how lateral occurrences can be eliminated even in the presence of bag promotion and bag difference, by means of a process we call *delateralization.*

Using the $\mathcal{NRC}_{\mathcal{G}}$ constructs, we can delateralize simple cases of deduplication or multiset difference as follows:

$$\begin{aligned}
&\biguplus\{\!|M \mid x \leftarrow N, y \leftarrow \iota(P)|\!\} \rightsquigarrow \biguplus\{\!|M \mid x \leftarrow N, y \leftarrow \iota(\mathcal{G}(x \leftarrow \delta N; P)) \circledast x|\!\} \\
&\biguplus\{\!|M \mid x \leftarrow N, y \leftarrow P_1 - P_2|\!\} \rightsquigarrow \\
&\qquad \biguplus\{\!|M \mid x \leftarrow N, y \leftarrow (\mathcal{G}(x \leftarrow \delta N; P_1) - \mathcal{G}(x \leftarrow \delta N; P_2)) \circledast x|\!\} \\
&\bigcup\{M \mid x \leftarrow N, y \leftarrow \delta(P_1 - P_2)\} \rightsquigarrow \\
&\qquad \bigcup\{M \mid x \leftarrow N, y \leftarrow \delta(\mathcal{G}(x \leftarrow N; P_1) - \mathcal{G}(x \leftarrow N; P_2)) \circledast x\}
\end{aligned}$$

It is necessary to deduplicate $N$ in the first two rules to ensure that the results correctly represent finite maps from the distinct elements of $N$ to multisets of corresponding elements of $P$. (In any case, $N$ needs to be deduplicated in order to be used as a set in $\mathcal{G}(x \leftarrow \delta N; \_)$).

Given a query expression in normal form, the above rules together with standard equivalences (such as commutativity of independent generators) can be used to delateralize it: that is, remove all occurrences of free variables in subexpressions of the form $\iota(N)$, $M_1 - M_2$, or $\delta(M_1 - M_2)$.

**Theorem 1.** *If* $M$ *is a flat query in normal form, then there exists* $M'$ *equivalent to* $M$ *with no lateral variable occurrences.*

The proof of correctness of the basic delateralization rules and the above correctness theorem are in the full version of this paper [44].

To illustrate some subtleties of the translation, here is a trickier example:

$$\biguplus \wr M \mid x \leftarrow N, y \leftarrow Q - \iota(P) \int$$

where $Q, P$ both depend on $x$. We proceed from the outside in, first delateralizing the difference:

$$\biguplus \wr M \mid x \leftarrow N, y \leftarrow (\mathcal{G}(x \leftarrow \delta(N); Q) - \mathcal{G}(x \leftarrow \delta(N); \iota(P))) \circledast x \int$$

Note that this still contains a lateral subquery, namely $\iota(P)$ depends on $x$. After translating back to $\mathcal{NRC}_\lambda(\mathit{Set}, \mathit{Bag})$, and delateralizing $\iota(P)$, the query normalizes to:

$$\begin{array}{l} Q_1 = \bigcup\{(x, z) \mid x \in \delta(N), z \leftarrow P\} \\ Q_2 = (\biguplus \wr (x, z) \mid x \in \iota\delta(N), z \leftarrow Q \int) - (\biguplus \wr (x, z) \mid x \in \iota\delta(N), (x', z) \leftarrow \iota(Q_1), x = x' \int) \\ \quad\quad \biguplus \wr M \mid x \leftarrow N, (x', y) \leftarrow Q_2, x = x' \int \end{array}$$

## 6 Query lifting and shredding

In the previous sections, we have discussed how to translate queries with flat collection input and output to SQL. The shredding technique, introduced in [8], can be used to convert queries with *nested* output (but flat input) to multiple flat queries that can be independently evaluated on an SQL database, then stitched together to obtain the required nested result. This section provides an improved version of shredding, extended to a more liberal setting mixing sets and bags and allowing bag difference operations, and described using the graph operations we have introduced, allowing an easier understanding of the shredding process.

We introduce, in Figure 6, a *shredding judgment* to denote the process by which, given a normalized $\mathcal{NRC}_\lambda(\mathit{Set}, \mathit{Bag})$ query, each of its subqueries having a nested collection type is lifted (in a manner analogous to lambda-lifting [30]) to an independent graph query: more specifically, shredding will produce a *shredding environment* (denoted by $\Phi, \Psi, \ldots$), which is a finite map associating special *graph variables* $\varphi, \psi$ to $\mathcal{NRC}_\mathcal{G}$ terms:

$$\Phi, \Psi, \ldots ::= [\overrightarrow{\varphi \mapsto M}]$$

The shredding judgment has the following form:

$$\Phi; \Theta \vdash M \mapsto \breve{M} \mid \Psi$$

where the $\mapsto$ symbol separates the input (to the left) from the output (to the right). The normalized $\mathcal{NRC}_\lambda(\mathit{Set}, \mathit{Bag})$ term $M$ is the query that is being considered for shredding; $M$ may contain free variables declared in $\Theta$, which must be a sequence of $\mathcal{NRC}_\lambda(\mathit{Set}, \mathit{Bag})$ set comprehension bindings. $\Theta$ is initially empty,

$$\frac{X \text{ is a base term}}{\Phi; \Theta \vdash X \mapsto X \mid \Phi} \qquad \frac{(\Phi_{i-1}; \Theta \vdash M_i \mapsto \breve{M}_i \mid \Phi_i)_{i=1,\ldots,n}}{\Phi_0; \Theta \vdash \langle \overrightarrow{\ell = M} \rangle \mapsto \langle \overrightarrow{\ell = \breve{M}} \rangle \mid \Phi_n}$$

$$\frac{\varphi \notin \mathrm{dom}(\Phi_n) \quad (\Phi_{i-1}; \Theta \vdash C_i \mapsto \psi_i \circledast \mathrm{dom}(\Theta) \mid \Phi_i)_{i=1,\ldots,n}}{\Phi_0; \Theta \vdash \bigcup \overrightarrow{C} \mapsto \varphi \circledast \mathrm{dom}(\Theta) \mid (\Phi_n \setminus \overrightarrow{\psi})[\varphi \mapsto \bigcup \overrightarrow{\Phi_n(\psi)}]}$$

$$\frac{\varphi \notin \mathrm{dom}(\Phi_n) \quad (\Phi_{i-1}; \Theta \vdash D_i \mapsto \psi_i \circledast \mathrm{dom}(\Theta) \mid \Phi_i)_{i=1,\ldots,n}}{\Phi_0; \Theta \vdash \biguplus \overrightarrow{D} \mapsto \varphi \circledast \mathrm{dom}(\Theta) \mid (\Phi_n \setminus \overrightarrow{\psi})[\varphi \mapsto \biguplus \overrightarrow{\Phi_n(\psi)}]}$$

$$\frac{\varphi \notin \mathrm{dom}(\Psi) \quad \Phi; \Theta, \overrightarrow{x \leftarrow F} \vdash M \mapsto \breve{M} \mid \Psi}{\Phi; \Theta \vdash \bigcup \{\{M\} \textbf{ where } X | \overrightarrow{x \leftarrow F}\} \mapsto \varphi \circledast \mathrm{dom}(\Theta) \mid \Psi[\varphi \mapsto \mathcal{G}(\Theta; \bigcup \{\{\breve{M}\} \textbf{ where } X | \overrightarrow{x \leftarrow F}\})]}$$

$$\frac{\varphi \notin \mathrm{dom}(\Psi) \quad \Phi; \Theta, \overrightarrow{x \leftarrow G^\delta} \vdash M \mapsto \breve{M} \mid \Psi}{\Phi_0; \Theta \vdash \biguplus \wr\wr M \wr \textbf{ where } X | \overrightarrow{x \leftarrow G} \wr \mapsto \varphi \circledast \mathrm{dom}(\Theta) \mid \Psi[\varphi \mapsto \mathcal{G}(\Theta; \biguplus \wr\wr \breve{M} \wr \textbf{ where } X | \overrightarrow{x \leftarrow G} \wr)]}$$

$$G^\delta \triangleq \begin{cases} Q^* & \text{if } G = \iota Q^* \\ \delta G & \text{otherwise} \end{cases} \qquad \Phi \setminus \overrightarrow{\psi} \triangleq [(\varphi \mapsto N) \in \Phi \mid \varphi \notin \overrightarrow{\psi}]$$

**Fig. 6.** Shredding rules.

but during shredding it is extended with parts of the input that have already been processed. Similarly, the input shredding environment $\Phi$ is initially empty, but will grow during shredding to collect shredded queries that have already been generated. It is crucial, for our algorithm to work, that $M$ be in the form previously described in Figure 3, as this allows us to make assumptions on its shape: in describing the judgment rules, we will use the same metavariables as are used in that grammar.

The output of shredding consists of a shredded term $\breve{M}$ and an output shredding environment $\Psi$. $\Psi$ extends $\Phi$ with the new queries obtained by shredding $M$; $\breve{M}$ is an output $\mathcal{NRC}_\mathcal{G}$ query obtained from $M$ by lifting its collection typed subqueries to independent queries defined in $\Psi$.

The rules for the shredding judgment operate as follows: the first rule expresses the fact that a normalized base term $X$ does not contain subexpressions with nested collection type, therefore it can be shredded to itself, leaving the shredding environment $\Phi$ unchanged; in the case of tuples, we perform shredding pointwise on each field, connecting the input and output shredding environments in a pipeline, and finally combining together the shredded subterms in the obvious way.

The shredding of collection terms (i.e. unions and comprehensions) is performed by means of *query lifting*: we turn the collection into a globally defined (graph) query, which will be associated to a fresh name $\varphi$ and instantiated to the local comprehension context by graph application. This operation is reminiscent

$$\frac{}{\vdash \cdot : \cdot} \qquad \frac{\vdash \Phi : \Gamma \qquad \Gamma \vdash M : \overrightarrow{\sigma} \multimap \tau \qquad \varphi \notin \mathrm{dom}(\Gamma)}{\vdash \Phi[\varphi \mapsto M] : (\Gamma, \varphi : \overrightarrow{\sigma} \multimap \tau)}$$

**Fig. 7.** Typing rules for shredding environments.

of the lambda lifting and closure conversion techniques used in the implementation of functional languages to convert local function definitions into global ones. Thus, when shredding a collection, besides processing its subterms recursively, we will need to extend the output shredding environment with a definition for the new global graph $\varphi$. In the interesting case of comprehensions, $\varphi$ is defined by graph-abstracting over the comprehension context $\Theta$; notice that, since we are only shredding normalized terms, we know that they have a certain shape and, in particular, the judgment for bag comprehensions must ensure that generators $\overrightarrow{G}$ be converted into sets.

The shredding of set and bag unions is performed by recursion on the subterms, using the same plumbing technique we employed for tuples; additionally, we optimize the output shredding environment by removing the graph queries $\overrightarrow{\psi}$ resulting from recursion, since they are absorbed into the new graph $\varphi$.

Notice that since the comprehension generators of our normalized queries must have a flat collection type, they do not need to be processed recursively. Furthermore, since our normal forms ensure that promotion and bag difference terms can only appear as comprehension generators, we do not need to provide rules for these cases.

The shredding environments used by the shredding judgment must be well typed, in the sense described by the rules of Figure 7: the judgment $\vdash \Phi : \Gamma$ means that the graph variables of $\Phi$ are mapped to terms whose type is described by $\Gamma$. Whenever we add a mapping $[\varphi \mapsto M]$ to $\Phi$, we must make sure that $M$ is well typed (of graph type) in the typing environment $\Gamma$ associated to $\Phi$.

If $\vdash \Phi : \Gamma$, we will write $ty(\Phi)$ to refer to the typing environment $\Gamma$ associated to $\Phi$. The following result states that shredding preserves well-typedness:

**Theorem 2.** *Let $\Theta$ be well-typed and $ty(\Theta) \vdash M : \sigma$. If $\Theta \vdash M \Mapsto \breve{M} \mid \Phi$, then:*

- *$\Phi$ is well-typed*
- *$ty(\Phi), ty(\Theta) \vdash \breve{M} : \sigma$*

We now intend to prove the correctness of shredding: first, we state a lemma which we can use to simplify certain expressions involving the semantics of graph application:

**Definition 2.** *Let $\Theta$ be a closed, well-typed sequence of generators. A substitution $\rho$ is a model of $\Theta$ (notation: $\rho \vDash \Theta$) if, and only if, for all $x \in \mathrm{dom}(\Theta)$, we have $[\![\Theta(x)]\!]\, \rho(x) > 0$.*

**Lemma 3.** *1.* $\left[\!\!\left[(\bigcup \overrightarrow{G}) \circledast (\overrightarrow{N})\right]\!\!\right] \rho = \bigvee_i \left[\!\!\left[G_i \circledast (\overrightarrow{N})\right]\!\!\right] \rho$

*2. If $\rho \vDash \Theta$, then for all $M$ we have $[\![\mathcal{G}(\Theta; M) \circledast (\mathrm{dom}(\Theta))]\!]\,\rho = [\![M]\!]\,\rho$.*

To state the correctness of shredding, we need the following notion of shredding environment substitution.

**Definition 3.** *For every well-typed shredding environment $\Phi$, the substitution of $\Phi$ into an $\mathcal{NRC_G}$ term $M$ (notation: $M\Phi$) is defined as the operation replacing within $M$ every free variable $\varphi \in \mathrm{dom}(\Phi)$ with $(\Phi(\varphi))\Phi$ (i.e.: the value assigned by $\Phi$ to $\varphi$, after recursively substituting $\Phi$).*

We can easily show that the above definition is well posed for well-typed $\Phi$.

We now show that shredding preserves the semantics of the input term, in the sense that the term obtained by substituting the output shredding environment into the output term is equivalent to the input.

**Theorem 3 (Correctness of shredding).** *Let $\Theta$ be well-typed and $ty(\Theta) \vdash M : \sigma$. If $\Phi; \Theta \vdash M \Mapsto \breve{M} \mid \Psi$, then, for all $\rho \vDash \Theta$, we have $[\![M]\!]\,\rho = [\![\breve{M}\Psi]\!]\,\rho$.*

*Proof.* By induction on the shredding judgment. We comment two representative cases:

– in the set comprehension case, we want to prove

$$[\![\textstyle\bigcup\{\{M\}\ \mathbf{where}\ X|\overrightarrow{x \leftarrow F}]\}]\!]\,\rho\ v = \\ [\![(\varphi \circledast (\mathrm{dom}(\Theta)))\Psi[\varphi \mapsto \textstyle\bigcup\{\mathcal{G}(\Theta; \textstyle\bigcup\{\{\breve{M}\}\ \mathbf{where}\ X|\overrightarrow{x \leftarrow F}\})\}]]\!]\,\rho\ v$$

where $\rho \vDash \Theta$. We rewrite the lhs as follows:

$$[\![\textstyle\bigcup\{\{M\}\ \mathbf{where}\ X|\overrightarrow{x \leftarrow F}]\}]\!]\,\rho\ v \\ = \textstyle\bigvee_{\overrightarrow{u}}([\![M]\!]\,\rho_n = v) \wedge ([\![X]\!]\,\rho_n) \wedge ([\![F_i]\!]\,\rho_{i-1}\ u_i))_{i=1,\dots,n}$$

where $\rho_i = \rho[x_1 \mapsto u_1, \dots, x_i \mapsto u_i] \vDash \Theta, x_1 \leftarrow F_1, \dots, x_i \leftarrow F_i$ for all $i = 1, \dots, n$, and $u_i$ s.t. $[\![F_i]\!]\,\rho_{i-1} u_i$. By the definition of substitution and by Lemma 3, we rewrite the rhs:

$$[\![(\varphi \circledast (\mathrm{dom}(\Theta)))\Psi[\varphi \mapsto \mathcal{G}(\Theta; \textstyle\bigcup\{\{\breve{M}\}\ \mathbf{where}\ X|\overrightarrow{x \leftarrow F}\})]]\!]\,\rho\ v \\ = [\![(\mathcal{G}(\Theta; \textstyle\bigcup\{\{\breve{M}\Psi\}\ \mathbf{where}\ X|\overrightarrow{x \leftarrow F}\})) \circledast (\mathrm{dom}(\Theta))]\!]\,\rho\ v \\ = [\![\textstyle\bigcup\{\{\breve{M}\Psi\}\ \mathbf{where}\ X|\overrightarrow{x \leftarrow F}\}]\!]\,\rho\ v \\ = \textstyle\bigvee_{\overrightarrow{u}}([\![\breve{M}\Psi]\!]\,\rho_n = v) \wedge ([\![F_i]\!]\,\rho_{i-1}\ u_i))_{i=1,\dots,n} \wedge ([\![X]\!]\,\rho')$$

We can prove that for all $\overrightarrow{u}$ such that $\rho_n \nvDash \Theta, \overrightarrow{x \leftarrow F}$, $([\![F_i]\!]\,\rho_{i-1}\ u_i)_{i=1,\dots,n} = 0$. Therefore, we only need to consider those $\overrightarrow{u}$ such that $\rho_n \vDash \Theta, \overrightarrow{x \leftarrow F}$. Then, to prove the thesis, we only need to show:

$$[\![M]\!]\,\rho_n = [\![\breve{M}\Phi]\!]\,\rho_n$$

which follows by induction hypothesis, for $\rho_n \vDash \Theta, \overrightarrow{x \leftarrow F}$.

– in the set union case, we want to prove

$$\left[\!\!\left[\bigcup \overrightarrow{C}\right]\!\!\right] \rho\ v = \left[\!\!\left[(\varphi \circledast (\mathrm{dom}(\Theta)))(\Psi \setminus \overrightarrow{\psi})[\varphi \mapsto \bigcup \overrightarrow{\Psi(\psi))}]\right]\!\!\right] \rho\ v$$

where $\rho \vDash \Theta$. We rewrite the lhs as follows:

$$\left[\!\!\left[\bigcup \overrightarrow{C}\right]\!\!\right] \rho\ v = \bigvee_i [\![C_i]\!]\ \rho\ v$$

By the definition of substitution and by Lemma 3, we rewrite the rhs:

$$\begin{aligned}
&\left[\!\!\left[(\varphi \circledast (\mathrm{dom}(\Theta)))(\Psi \setminus \overrightarrow{\psi})[\varphi \mapsto \bigcup \overrightarrow{\Psi(\psi))}]\right]\!\!\right] \rho\ v \\
&= \left[\!\!\left[(\bigcup \overrightarrow{(\Psi(\psi))\Psi}) \circledast (\mathrm{dom}(\Theta))\right]\!\!\right] \rho\ v \\
&= \textstyle\bigvee_i [\![(\Psi(\psi_i))\Psi \circledast (\mathrm{dom}(\Theta))]\!]\ \rho\ v
\end{aligned}$$

By induction hypothesis and unfolding of definitions, we know for all $i$:

$$[\![C_i]\!]\ \rho = \left[\!\!\left[(\psi_i \circledast (\overrightarrow{\mathrm{dom}(\Theta)}))\Psi\right]\!\!\right] \rho = \left[\!\!\left[(\Psi(\psi_i))\Psi \circledast (\overrightarrow{\mathrm{dom}(\Theta)})\right]\!\!\right] \rho$$

which proves the thesis. □

### 6.1 Reflecting shredded queries into $\mathcal{NRC}_\lambda(Set, Bag)$

The output of the shredding judgment is a stratified version of the input term, where each element of the output shredding environment provides a layer of collection nesting; furthermore, the output is ordered so that each element of the shredding environment only references graph variables defined to its left, which is convenient for evaluation. Our goal is to evaluate each shredded item as an independent query: however, these items are not immediately convertible to flat queries, partly because their type is still nested, and also due to the presence of graph operations introduced during shredding. We thus need to provide a translation operation capable of converting the output of shredding into independent flat terms of $\mathcal{NRC}_\lambda(Set, Bag)$. This translation uses two main ingredients:

– an *index* function to convert graph variable references to a flat type $\mathbb{I}$ of indices, such that $\phi, \overrightarrow{x}$ are recoverable from $index(\phi, \overrightarrow{x})$;
– a technique to express graphs as standard $\mathcal{NRC}_\lambda(Set, Bag)$ relations.

The resulting translation, denoted by $\lfloor\cdot\rfloor$, is shown in in Figure 8. Let us remark that the translation need be defined only for term forms that can be produced as the output of shredding: this allows us, for instance, not to consider terms such as $\iota M$ or $M - N$, which can only appear as part of flat generators of comprehensions or graphs.

We discuss briefly the interesting cases of the definition of the flattening translation. Base expressions $X$ are expressible in $\mathcal{NRC}_\lambda(Set, Bag)$, therefore they can be mapped to themselves (this is also true for **empty**$(M)$, since normalization ensures that the type of $M$ be a flat collection). Graph applications

$$\lfloor X \rfloor = X \qquad \left\lfloor \langle \overrightarrow{\ell = M} \rangle \right\rfloor = \langle \overrightarrow{\ell = \lfloor M \rfloor} \rangle$$

$$\left\lfloor \bigcup \overrightarrow{C} \right\rfloor = \bigcup \overrightarrow{\lfloor C \rfloor} \qquad \left\lfloor \biguplus \overrightarrow{D} \right\rfloor = \biguplus \overrightarrow{\lfloor D \rfloor}$$

$$\lfloor \varphi \circledast (\overrightarrow{x}) \rfloor = index(\varphi, \overrightarrow{x})$$

$$\left\lfloor \bigcup \{\{M\} \textbf{ where } X | \overrightarrow{x \leftarrow F}\} \right\rfloor = \bigcup \{\{\lfloor M \rfloor\} \textbf{ where } X | \overrightarrow{x \leftarrow F}\}$$

$$\left\lfloor \biguplus \wr\wr M \wr \textbf{ where } X | \overrightarrow{x \leftarrow G} \wr \right\rfloor = \biguplus \wr\wr \lfloor M \rfloor \wr \textbf{ where } X | \overrightarrow{x \leftarrow G} \wr$$

$$\left\lfloor \mathcal{G}^{\mathsf{set}}(\overrightarrow{x \leftarrow F}; M) \right\rfloor = \bigcup \{\langle x, y \rangle | \overrightarrow{x \leftarrow F}, y \leftarrow \lfloor M \rfloor\}$$

$$\left\lfloor \mathcal{G}^{\mathsf{bag}}(\overrightarrow{x \leftarrow F}; M) \right\rfloor = \biguplus \wr \langle x, y \rangle | \overrightarrow{x \leftarrow \iota F}, y \leftarrow \lfloor M \rfloor \wr$$

**Fig. 8.** Flattening embedding of shredded queries into $\mathcal{NRC}_\lambda(Set, Bag)$.

$\varphi \circledast (\overrightarrow{x})$, as we said, are translated with the help of an *index* abstract operation: this is where the primary purpose of the translation is accomplished, by flattening a collection type to the flat type $\mathbb{I}$, making it possible for a shredded query to be converted to SQL; although we do not specify the concrete implementation of *index*, it is worth noting that it must store the arguments of the graph application along with the (quoted) *name* of the graph variable $\varphi$. Tuples, unions, and comprehensions only require a recursive translation of their subterms: however the generators of comprehensions must have a flat collection type, so no recursion is needed there. Finally, we translate graphs as collections of the pairs obtained by associating elements of the domain of the graph to the corresponding output; it is simple to come up with a comprehension term building such a collection: set-valued graphs are translated using set comprehension, while bag-valued ones use bag comprehension (this also means that in the latter case the generators for the domain of the graph, which are set-typed, must be wrapped in a $\iota$).

We can prove that the flattening embedding produces flat-typed terms, as expected.

**Definition 4.** *A well-typed set comprehension generator $\Theta$ is flat-typed if, and only if, for all $x \in \mathrm{dom}(\Theta)$, there exists a flat type $\sigma$ such that $ty(\Theta(x)) = \{\sigma\}$.*

*A well-typed shredding environment $\Phi$ is flat-typed if, and only if, for all $\varphi \in \mathrm{dom}(\Phi)$, we have that $ty(\lfloor \Phi(\varphi) \rfloor)$ is a flat collection type.*

**Lemma 4.** *Suppose $\Phi; \Theta \vdash M \mapsto \breve{M} \mid \Psi$, where $\Phi$ and $\Theta$ are flat-typed. Then, $\breve{M}$ and $\Psi$ are also flat-typed.*

It is important to note that the composition of shredding and $\lfloor \cdot \rfloor$ does not produce normalized $\mathcal{NRC}_\lambda(Set, Bag)$ terms: when we shred a comprehension, we add to the output shredding environment a graph returning a comprehension, and when we translate this to $\mathcal{NRC}_\lambda(Set, Bag)$ we get two nested comprehensions:

$$\left\lfloor \mathcal{G}(x \leftarrow \delta t; \biguplus \wr\wr \breve{M} \wr | y \leftarrow \iota Q^* \wr) \right\rfloor = \biguplus \wr \langle x, z \rangle | x \leftarrow \iota \delta t, z \leftarrow \biguplus \wr\wr \left\lfloor \breve{M} \right\rfloor \wr | y \leftarrow \iota Q^* \wr\wr$$

$$(\!|X : b|\!)\Xi \triangleq X \qquad \text{(if } X \text{ is not an index)}$$
$$(\!|\langle\overrightarrow{\ell = N}\rangle : \langle\overrightarrow{\ell : \tau}\rangle|\!)\Xi \triangleq \langle\overrightarrow{\ell = (\!|\breve{N} : \tau|\!)\Xi}\rangle$$
$$(\!|\langle\overrightarrow{\ell = N}\rangle.\ell_i : \tau|\!)\Xi \triangleq (\!|N_i : \tau|\!)\Xi$$
$$(\!|index(\varphi, \overrightarrow{V}) : \{\tau\}|\!)\Xi \triangleq \bigcup\{\{(\!|p.2 : \tau|\!)\Xi\} \mid p \leftarrow \Xi(\varphi), p.1 = \langle\overrightarrow{V}\rangle\}$$
$$(\!|index(\varphi, \overrightarrow{V}) : \wr\tau\wr|\!)\Xi \triangleq \biguplus\wr\wr(\!|p.2 : \tau|\!)\Xi\wr \mid p \leftarrow \Xi(\varphi), p.1 = \langle\overrightarrow{V}\rangle\wr$$

**Fig. 9.** The stitching function.

In fact, not only is this term not in normal form, but it may even contain, within $Q^*$, a lateral reference to $x$; thus, after a flattening translation, we will always require the resulting queries to be renormalized and, if needed, delateralized.

Let *norm* denote $\mathcal{NRC}_\lambda(Set, Bag)$ normalization, and $\mathcal{S}$ denote the evaluation of relational normal forms: we define the shredded value set $\Xi$ corresponding to a shredding environment $\Phi$ as follows:

$$\Xi \triangleq \{\varphi \mapsto \mathcal{S}(norm(\lfloor M \rfloor)) | [\varphi \mapsto M] \in \Phi\}$$

The evaluation $\mathcal{S}$ is ordinarily performed by a DBMS after converting the $\mathcal{NRC}_\lambda(Set, Bag)$ query to SQL, as described in Section 5. The result of this evaluation is reflected in a programming language such as Links as a list of records.

### 6.2 The stitching function

Given a $\mathcal{NRC}_\lambda(Set, Bag)$ term with nested collections, we have first shredded it, obtaining a shredded $\mathcal{NRC}_\mathcal{G}$ term $\breve{M}$ and a shredding environment $\Phi$ containing $\mathcal{NRC}_\mathcal{G}$ graphs; then we have used a flattening embedding to reflect both $\breve{M}$ and $\Phi$ back into the flat fragment of $\mathcal{NRC}_\lambda(Set, Bag)$; next we used normalization and DBMS evaluation to convert the shredding environment into a shredded value set $\Xi$. As the last step to evaluate $M : \tau$, we need to combine $\left\lfloor \breve{M} \right\rfloor$ and $\Xi$ together to reconstruct the correct nested value $(\!|\left\lfloor \breve{M} \right\rfloor : \tau|\!)\Xi$ by *stitching* together partial flat values.

The stitching function is shown in Figure 9: its job is to visit all the components of tuples and collections, ignoring atomic values other than indices along the way. The real work is performed when an $index(\varphi, \overrightarrow{V})$ is found: conceptually, the index should be replaced by the result of the evaluation of $\varphi \circledast (\overrightarrow{V})$. Remember that $\Xi$ contains the result of the evaluation of the graph function $\varphi$ after translation to $\mathcal{NRC}_\lambda(Set, Bag)$, i.e. a collection of pairs associating each input of $\varphi$ to the corresponding output: then, to obtain the desired result, we can take $\Xi(\varphi)$, filter all the pairs $p$ whose first component is $\langle\overrightarrow{V}\rangle$, and return the second component of $p$ after a recursive stitching. Finally, observe that we track the result type argument in order to disambiguate whether to construct a set or multiset when we encounter an index.

**Theorem 4 (Correctness of stitching).** *Let $\Theta$ be well-typed and $ty(\Theta) \vdash M : \sigma$. Let $\Phi$ be well-typed, and suppose $\Phi; \Theta \vdash M \Rightarrow \breve{M} \mid \Psi$. Let $\Xi$ be the result of evaluating the flattened queries in $\Psi$ as above. Then $\left[\!\left[ \breve{M}\Psi \right]\!\right] \rho = \left[\!\left[ (\!| \left\lfloor \breve{M} \right\rfloor : \tau |\!) \Xi \right]\!\right] \rho$.*

The full correctness result follows by combining the Theorems 3 and 4.

**Corollary 1.** *For all $M$ such that $\vdash M : \tau$, suppose $\vdash M \Rightarrow \breve{M}' \mid \Psi$, and let $\Xi$ be the shredded value set obtained by evaluating the flattened queries in $\Psi$. Then $[\![M]\!] = \left[\!\left[ (\!| \left\lfloor \breve{M} \right\rfloor : \tau |\!) \Xi \right]\!\right]$.*

## 7 Related work

Work on language-integrated query and comprehension syntax has taken place over several decades in both the database and programming language communities. We discuss the most closely related work below.

*Comprehensions, normalization and language integration* The database community had already begun in the late 1980s to explore proposals for so-called *non-first-normal-form* relations in which collections could be nested inside other collections [46], but following Trinder and Wadler's initial work connecting database queries with monadic comprehensions [50], query languages based on these foundations were studied extensively, particularly by Buneman et al. [4,3]. For our purposes, Wong's work on query normalization and translation to SQL [55] is the most important landmark; this work provided the basis for practical implementations such as Kleisli and later Links. Almost as important is the later work by Libkin and Wong [33], studying the questions of expressiveness of bag query languages via a language $\mathcal{BQL}$ that extended basic $\mathcal{NRC}$ with deduplication and bag difference operators. They related this language to $\mathcal{NRC}$ with set semantics extended with aggregation (count/sum) operations, but did not directly address the question of normalizing and translating $\mathcal{BQL}$ queries to SQL. Grust and Scholl [28] were early advocates of the use of comprehensions mixing set, bag and other monadic collections for query rewriting and optimization, but did not study normalization or translatability properties.

Although comprehension-based queries began to be used in general-purpose programming languages with the advent of Microsoft LINQ [36] and Links [12], Cooper [11] made the next important foundational contribution by extending Wong's normalization result to queries containing higher-order functions and showing that an effect system could be used to safely compose queries using higher-order functions even in an ambient language with side-effects and recursive functions that cannot be used in queries. This work provided the basis for subsequent development of language-integrated query in Links [34] and was later adapted for use in F# [7], Scala [41], and by Kiselyov et al. [48] in the OCaml library QUE$\Lambda$. However, on revisiting Cooper's proof to extend it to heterogeneous queries, we found a subtle gap in the proof, which was corrected in a recent paper [43]; the original result was correct. As a result, in this paper we focus on first-order fragments of these languages without loss of generality.

Giorgidze et al. [22] have shown how to support non-recursive datatypes (i.e. sums) and Grust and Ulrich [29] built on this to show how to support function types in query results using defunctionalization [29]. We considered using sums to support a defunctionalization-style strategy for query lifting, but Giorgidze et al. [22] map sum types to nested collections, which makes their approach unsuitable to our setting. Wong's original normalization result also considered sum types, but to the best of our knowledge normalization for $\mathcal{NRC}_\lambda(Set, Bag)$ extended with sum types has not yet been proved.

Recent work by Suzuki et al. [48] have outlined further extensions to language-integrated query in the QUE$\Lambda$ system, which is based on finally-tagless syntax [6] and employs Wong's and Cooper's rewrite rules; Katsushima and Kiselyov's subsequent short paper [31] outlined extensions to handling ordering and grouping. Kiselyov and Katsushima [32] present an extension to QUE$\Lambda$ called SQUR to handle ordering based on effect typing, and they provide an elegant translation from SQUR queries to SQL based on normalization-by-evaluation. Okura and Kameyama [39] outline an extension to handle SQL-style grouping and aggregation operators in QUE$\Lambda_G$; however, their approach potentially generates lateral variable occurrences inside grouping queries. These systems QUE$\Lambda$, SQUR and QUE$\Lambda_G$ consider neither heterogeneity nor nested results.

Our adoption of tabulated functions (*graphs*) is inspired in part by Gibbons et al. [20], who provided an elegant rational reconstruction of relational algebra showing how standard principles for reasoning about queries arise from adjunctions. They employed types for (finite) maps and tables to show how joins can be implemented efficiently, and observed that such structures form a *graded monad*. We are interested in further exploring these structures and extending our work to cover ordering, grouping and aggregation.

*Query decorrelation and delateralization* There is a large literature on *query decorrelation*, for example to remove aggregation operations from `SELECT` or `WHERE` clauses (see e.g. [38,5] for further discussion). Delateralization appears related to decorrelation, but we are aware of only a few works on this problem, perhaps because most DBMSs only started to support `LATERAL` in the last few years. (Microsoft SQL Server has supported similar functionality for much longer through a keyword `APPLY`.) Our delateralization technique appears most closely related to Neumann and Kemper's work on query unnesting [38]. In this context, unnesting refers to removal of "dependent join" expressions in a relational algebraic query language; such joins appear to correspond to lateral subqueries. This approach is implemented in the HyPER database system, but is not accompanied by a proof of correctness, nor does it handle nested query results. It would be interesting to formalize this approach (or others from the decorrelation literature) and relate it to delateralization.

*Querying nested collections* Our approach to querying nested heterogeneous collections clearly specializes to the homogeneous cases for sets and multisets respectively, which have been studied separately. Van den Bussche's work on simulating queries on nested sets using flat ones [54] has also inspired subsequent work on query shredding, flattening and (in this paper) lifting, though the simulation technique itself does not appear practical (as discussed in the extended version of Cheney et al. [9]). More recently, Benedikt and Pradic [1] presented results on representing queries on nested collections using a bounded number of *interpretations* (first-order logic formulas corresponding to definable flat query expressions) in the context of their work on *synthesizing* $\mathcal{NRC}$ queries from proofs. This approach considers set-valued $\mathcal{NRC}$ only, and its relationship to our approach should be investigated further.

Cheney et al.'s previous work on query shredding for multiset queries [8] is different in several important respects. In that work we did not consider deduplication and bag difference operations from $\mathcal{BQL}$, which Libkin and Wong showed cannot be expressed in terms of other $\mathcal{NRC}$ operations. The shredding translation was given in several stages, and while each stage is individually comprehensible, the overall approach is not easy to understand. Finally, the last stages of the translation relied on SQL features not present (or expressible) in the source language, such as ordering and the SQL:1999 `ROW_NUMBER` construct, to synthesize uniform integer keys. Our approach, in contrast, handles set, bag, and mixed queries, and does not rely on any SQL:1999 features.

In a parallel line of work, Grust et al. [26,21,51,53,52] have developed a number of approaches to querying nested *list* data structures, first in the context of XML processing [24] and subsequently for $\mathcal{NRC}$-like languages over lists. The earlier approach [26], named *loop-lifting* (not to be confused with *query lifting*!) made heavy use of SQL:1999 capabilities for numbering and indexing to decouple nested collections from their context, and was implemented in both Links [51] and earlier versions of the Database Supported Haskell library [21], both of which relied on an advanced query optimizer called *Pathfinder* [27] to optimize these queries. The more recent approach, implemented by Ulrich in the current version of DSH and described in detail in his thesis [52], is called *query flattening* and is instead based on techniques from *nested data parallelism* [2]. Both loop-lifting and query flattening are very powerful, and do not rely on an initial normalization stage, while supporting a rich source language with list semantics, ordering, grouping, aggregation, and deduplication which can in principle emulate set or multiset semantics. However, to the best of our knowledge no correctness proofs exist for either technique. We view finding correctness results for richer query languages as an important challenge for future work.

Another parallel line of work started by Fegaras and Maier [15,14] considers heterogeneous query languages based on *monoid* comprehensions, with set, list, and bag collections as well as grouping, aggregation and ordering operations, in the setting of object-oriented databases, and forms the basis for complex object database systems such as $\lambda$DB [16] and Apache MRQL [14]. However, Wong-style normalization results or translations from flat or nested queries to SQL are not known for these calculi.

*Lambda-lifting and closure conversion* Since Johnsson's original work [30], lambda-lifting and closure conversion have been studied extensively for functional languages, with Minamide et al.'s *typed closure conversion* [37] of particular interest in compilers employing typed intermediate languages. We plan to study whether known optimizations in the lambda-lifting and closure conversion literature offer advantages for query lifting. The immediate important next step is to implement our approach and compare it empirically with previous techniques such as query shredding and query flattening. By analogy with lambda-lifting and closure conversion, we expect additional optimizations to be possible by a deeper analysis of how variables/fields are used in lifted subqueries. Another problem we have not resolved is how to deal with deduplication or bag difference at nested collection types in practice. Libkin and Wong [33] showed that such nesting can be eliminated from $\mathcal{BQL}$ queries, but their results do not provide a constructive algorithm for eliminating the nesting.

## 8 Conclusions

Monadic comprehensions have proved to be a remarkably durable foundation for database programming and language-integrated query, and has led to language support (LINQ for .NET, Quill for Scala) with widespread adoption. Recent work has demonstrated that techniques for evaluating queries over nested collections, such as query shredding or query flattening, can offer order-of-magnitude speedups in database applications [19] without sacrificing declarativity or readability. However, query shredding lacks the ability to express common operations such as deduplication, while query flattening is more expressive but lacks a detailed proof of correctness, and both techniques are challenging to understand, implement, or extend. We provide the first provably correct approach to querying nested heterogeneous collections involving both sets and multisets.

Our most important insight is that working in a heterogeneous language, with both set and multiset collection types, actually makes the problem easier, by making it possible to calculate finite maps representing the behavior of nested query subexpressions under all of the possible environments encountered at run time. Thus, instead of having to maintain or synthesize keys linking inner and outer collections, as is done in all previous approaches, we can instead use the values of variables in the closures of nested query expressions themselves as the keys. The same approach can be used to eliminate sideways information-passing. This is analogous to lambda-lifting or closure conversion in compilation of functional languages, but differs in that we lift local queries to (queries that compute) finite maps rather than ordinary function abstractions. We believe this idea may have broader applications and will next investigate its behavior in practice and applications to other query language features.

*Acknowledgments* This work was supported by ERC Consolidator Grant Skye (grant number 682315), and by an ISCF Metrology Fellowship grant provided by the UK government's Department for Business, Energy and Industrial Strategy (BEIS). We are grateful to Simon Fowler for feedback and to anonymous reviewers for constructive comments.
